# Genetic Testing for Rare Diseases

**DOI:** 10.3390/diagnostics12040809

**Published:** 2022-03-25

**Authors:** José M. Millán, Gema García-García

**Affiliations:** 1 Instituto de Investigación Sanitaria La Fe, Molecular, Cellular and Genomics Biomedicine, 46026 Valencia, Spain; gema.gegargar@gmail.com; 2 Centro de Investigación Biomédica en Red de Enfermedades Raras (CIBERER), 28029 Madrid, Spain

The term rare disease was coined in the 1970s to refer to diseases that have a low prevalence. However, the definition varies among countries. The European Commission defines a rare disease as a disease that affects less than 5 in 10,000 people. In the US, a global number of cases is used (less than 200,000 cases for the entire country) [1]. Other countries use a more restrictive definition, such as less than 4 cases per 10,000 in Japan or even less than 2 cases per 10,000 in other countries [2].

Beyond the prevalence, the definition of a rare disease must include other issues, such as chronic and severe disorders that usually have an early onset but can begin in adulthood; diseases that can affect every organ or even different organs; diseases that are not well-understood and lack information about them; diseases that do not have a treatment or only a treatment that is not very effective.

It is estimated that there are 6000–8000 diseases included in this denomination [1]. They are tremendously heterogeneous, and about 80% are genetic (often monogenic) [3].

Even though a single rare disease affects only a few patients, the high number of rare diseases means they affect about 3.5–6% of all people globally, which is between 263 and 446 million people [4].

In summary, the definition of the term rare disease, besides the prevalence, must see them as a wide and varied group of disorders that each affect a small number of persons, which are chronic and disabling and have a high rate of morbi-mortality, with scarce and limited therapeutical resources [5].

The absence of a diagnosis (or perhaps a correct diagnosis) can have serious consequences for the patients and their relatives. Additionally, the heterogeneity of national capabilities regarding genetic testing (and changing technologies for such testing) may impact the access to diagnosis. The diagnosis of some rare diseases may delay even five years.

Delays in the diagnosis may cause further aggravation of the disease, inadequate treatments, lack of treatments or support, and the possibility of recurrence in the family as most rare diseases are genetic and the absence of diagnosis has prevented successful genetic counseling [6].

In this sense, the International Rare Diseases Research Consortium, IRDiRC, has marked as a key objective to improve the time of diagnosis and the accessibility to it. A new general vision was adopted for the period 2017–2027: “To make it possible for all people suffering from a rare disease to receive an accurate diagnosis, care and available therapy within one year of seeking medical assistance.” [7].

To turn this vision into a reality, three new goals were agreed upon:

Goal 1: All patients coming to medical attention with a suspected rare disease will be diagnosed within one year if their disorder is known in the medical literature; all currently undiagnosable individuals will enter a globally coordinated diagnostic and research pipeline;
Goal 2: A total of 1000 new therapies for rare diseases will be approved, the majority of which will focus on diseases without approved options;
Goal 3: Methodologies will be developed to assess the impact of diagnoses and therapies on rare disease patients.

An increment of medical products for rare diseases based on gene therapy can be foreseen. Spinal muscular atrophy is a good example of how gene therapy can change the nature of a devastating disease. The good prognosis of SMA with early administration of the available medical products recently approved by the FDA and EMA has boosted the implementation of SMA newborn screening in several countries [8,9].

However, there are other examples of gene-based therapeutical approaches approved by medical agencies or in clinical trials.

All the information mentioned above makes the (as earlier as possible) genetic diagnoses of rare diseases essential to apply these therapies to the right patients.

In this Special Issue, there are several interesting examples of genetic diagnosis of rare diseases that affect different organs and tissues.

Boutouchent et al. [10] describe a case report of an atypical late-onset patient with 3-Hydroxy-3-methylglutaryl-CoA Lyase Deficiency (HMGLD). A 54-year-old female was suspicious of HMGLD, although this disease usually has its onset in the first few months of life. They sequenced the HMGCL gene and found two variants, one of them previously described as pathogenic and the other one was not reported before but was predicted to skip the exon 1. These variants would explain the disease. The late onset of the disease, in this case, led to it being undiagnosed for years, and the integrative interpretation of imaging, biochemical, and molecular findings enabled the authors to reach the diagnosis of this treatable condition.

The genetic diagnosis allows correct genetic counseling. This is key for patients to plan their professional lives and to choose among the different reproductive options. Álvaro-Sánchez et al. [11] evaluate the current situation in which rare disease patients receive genetic services in Spain. The Spanish laws state that genetic counselling is mandatory before and after the genetic test, but, surprisingly, there is a lack of recognition in Spain of Clinical Genetics as a healthcare specialty). They provide a comprehensive review of the number of centers (public and private) and their distribution among the different regions. They conclude that the lack of specialty makes it difficult to implement genetic counselling in Spain and that the Clinical Genetics specialty urgently needs to be recognized to provide a multidisciplinary service to patients with rare diseases.

Danilchenko et al. [12] reported the prevalence of SLC26A4 among patients with hearing impairment in two different South Siberian populations: Tuvinians and Altaians. With this and a previous study, they were able to uncover the genetic causes of hearing loss in 50.5% and 34.5% of Tuvinian and Altaian patients, respectively, expanding the landscape of the genetics underlying the hearing loss in two understudied populations.

An additional case report concerning hearing loss is reported by Cenni et al. [13]. They report a large family in which thrombocytopenia, post-lingual hearing loss, and congenital hearing loss coexist. After a hearing loss panel sequencing and whole-exome sequencing, they found a pathogenic variant in MHY9 that explains the thrombocytopenia, a mutation in MYO7A that explains the post-lingual hearing loss and a de novo mutation in a child responsible for the congenital hearing loss. This family illustrates not only the issue of the coexistence of several rare diseases in a single family but also the presence of several mutated genes for a single medical condition (in this case, hearing loss) genetically heterogeneous.

Sival et al. [14] conducted a comprehensive multidisciplinary study on 80 patients with Early adult Onset Ataxia (EOA) with and without dystonic comorbidity. They found that comorbid dystonia is present in the majority of the EOA patients. They found mutations in genes involved in pathways, such as energy depletion and signal transduction in the cortical–basal–ganglia–pontine–cerebellar network.

Another manuscript about the genetics in hearing impairment in this Special Issue is the one by Mansard et al. [15]. Their study of two unrelated families with autosomal dominant non-syndromic hearing loss identified for the first time one copy number variant in the exon 8 of the *GSDME* gene in each family. They remark the importance of a comprehensive analysis of copy number variants for genetic diagnosis.

Barp et al. [16] conducted a review about the complexity of the molecular diagnosis of neuromuscular disorders, including gene panels sequencing, whole-exome sequencing, and whole-genome sequencing. They highlight the importance of clinical diagnoses in order to target the appropriate technique and candidate genes according to the suspected clinical entity and the challenge that supposes the pathogenic nature of a high number of variants of unknown significance that are found with the use of next-generation sequencing.

Lecka-Ambroziak et al. [17] reported a genotype–phenotype correlation in a cohort of 147 Polish patients with Prader-Willi syndrome, stratifying them according to the genetic defect that causes the disease and the importance of the time of diagnosis before the commencement of the recombinant human growth hormone treatment.

Park and colleagues [18] report a case of a 13-year-old female with mosaic Turner syndrome and complete growth hormone deficiency and pituitary microadenoma. They also make an excellent review of the literature and compare all the cases of Turner syndrome and growth hormone deficiency and of Turner syndrome and pituitary microadenoma reported to date.

Sudrié-Arnaud et al. [19] designed a custom gene panel sequencing including 51 genes responsible for lysosomal disorders and validated it in 21 well-characterized patients. The bioinformatic pipelines used were also validated to detect single nucleotide variants, copy number variants and indels. Furthermore, they validated the panel in five new cases.

Finally, Tatur and Ben-Yosef [20] review the clinical entities and genetics of over 80 syndromic inherited retinal dystrophies, highlighting the percentage of organs/tissues involved in these syndromes apart of the retina.

In summary, this Special Issue shows a wide variety of rare diseases and the great advances that next-generation sequencing has supposed genetically diagnosing them.
